# Prader-Willi Syndrome and Schaaf-Yang Syndrome: Neurodevelopmental Diseases Intersecting at the *MAGEL2* Gene

**DOI:** 10.3390/diseases4010002

**Published:** 2016-01-13

**Authors:** Michael D. Fountain, Christian P. Schaaf

**Affiliations:** 1 Interdepartmental Program in Translational Biology and Molecular Medicine, Department of Molecular and Human Genetics, Baylor College of Medicine, Houston, TX 77030, USA; mfountai@bcm.edu; 2 Jan and Dan Duncan Neurological Research Institute, Texas Children’s Hospital, Houston, TX 77030, USA

**Keywords:** Prader-Willi syndrome, Schaaf-Yang syndrome, MAGEL2, USP7, neurodevelopmental disorders

## Abstract

Prader-Willi syndrome (PWS) is a neurodevelopmental disorder characterized by neonatal hypotonia, developmental delay/intellectual disability, and characteristic feeding behaviors with failure to thrive during infancy; followed by hyperphagia and excessive weight gain later in childhood. Individuals with PWS also manifest complex behavioral phenotypes. Approximately 25% meet criteria for autism spectrum disorder (ASD). PWS is caused by the absence of paternally expressed, maternally silenced genes at chromosome 15q11-q13. *MAGEL2* is one of five protein-coding genes in the PWS-critical domain. Truncating point mutations of the paternal allele of *MAGEL2* cause Schaaf-Yang syndrome, which has significant phenotypic overlap with PWS, but is also clinically distinct; based on the presence of joint contractures, and a particularly high prevalence of autism spectrum disorder (up to 75% of affected individuals). The clinical and molecular overlap between PWS and Schaaf-Yang syndrome, but also their distinguishing features provide insight into the pathogenetic mechanisms underlying both disorders.

## 1. Introduction

Prader-Willi syndrome (PWS) is a complex genetic disorder caused from alterations of the paternally expressed genes on chromosome 15q11-q13 [1]. In the early years of life, individuals with PWS manifest severe hypotonia, feeding difficulties, failure to thrive, and developmental delays [2]. Hypogonadotropic hypogonadism is present, most readily appreciated in boys due to undescended testicles and a hypoplastic phallus. Following the marked feeding difficulties during infancy, children with PWS will then go on to develop hyperphagia, leading to morbid obesity unless diet is restricted [1,2]. As the children get older, a typical cognitive and behavioral profile becomes evident. Most are affected with intellectual disability. Their behavior is comprised of stubbornness, temper tantrums, manipulative and compulsive behaviors [3,4]. Strabismus, scoliosis, and sleep disturbances are also noted. Characteristic facial dysmorphisms include a narrowed bifrontal diameter, almond-shaped palpebral fissures, a narrow nasal bridge, and down-turned corners of the mouth, which may be apparent at birth and/or can manifest with time [1,2].

The PWS critical region lies within a 6 Mb genomic locus on the long arm of chromosome 15. The two primary molecular causes of PWS include deletion of paternal 15q11-q13, which is present in 65%–75% of individuals with PWS, and maternal uniparental disomy, present 20%–30% of cases. Defects of the imprinting center are observed in 1%–3% of cases [3,5,6,7]. The maternally imprinted, paternally expressed genes included in the PWS-critical region are *MKRN3*, *MAGEL2*, *NDN*, *NPAP1*, *SNURF-SNRPN*, and a family of six small nucleolar RNA (snoRNA) genes [1,7].

While the vast majority of individuals with PWS have loss of expression of all of these genes, a small number of cases have been reported that may help elucidate which genes account for some of the phenotypes characteristic of PWS. A lot of interest has focused on the region containing the *SNORD116* snoRNA cluster, as its loss appears to account for several key phenotypes observed in PWS. An individual with microdeletion of the *SNORD116* cluster was reported to manifest neonatal hypotonia, feeding difficulties and failure to thrive, then hyperphagia leading to morbid obesity, hypogonadism, developmental delay, and some PWS facial features [8]. In addition, this individual met criteria for autism spectrum disorder (ASD), based on the Autism Diagnostic Observation Schedule (ADOS) and Autism Diagnostic Interview-Revised (ADI-R). Subsequently, two additional cases were reported with overlapping microdeletions including *SNORD116*, and both manifested similar clinical features, including hyperphagia and obesity [9,10].

## 2. The *MAGEL2* Gene in Prader-Willi Syndrome and Schaaf-Yang Syndrome

More recently, a series of individuals with point mutations in a protein-coding gene of the PWS domain was reported. Whole genome and whole exome sequencing identified four individuals with truncating pathogenic variants in *MAGEL2*. These initial individuals manifested a phenotypic spectrum of neonatal hypotonia, feeding difficulties, weight gain, developmental delay/intellectual disability, and hypogonadism [11]. As neonates, all were suspected to have PWS, but standard molecular diagnostic testing, which is based on abnormal methylation patterns of genomic DNA at 15q11-q13, was negative. Two of the four individuals manifested contractures of the finger joints. All four were clinically assessed and diagnosed with ASD based on DSM-IV criteria. Interestingly, these individuals tended to lack the extreme hyperphagia, and subsequently did not develop the morbid obesity that is typically seen in untreated PWS as evidenced by the calculated body mass indices (Table 1). This suggested a disease entity similar to PWS, yet distinct, and was subsequently named Schaaf-Yang syndrome (OMIM 615547).

**Table 1:** Height, weight, and BMI of reported individuals with truncating point mutations or whole gene deletions of *MAGEL2*.

Patient	Mutation Type	Age	Sex	Height	Height Z-Score	Weight	Weight Z-Score	BMI	BMI Z-Score
Schaaf *et al.* subject 1	Truncating point mutation	13 yo	M	156 cm	−0.03	54.2 kg	0.82	22.3	1.13
Schaaf *et al.* subject 2	Truncating point mutation	7 yr 6 mo	M	134 cm	1.65	68.3 kg	3	38	2.99
Schaaf *et al.* subject 3	Truncating point mutation	5 yo	M	105 cm	−0.91	19.6 kg	0.42	17.8	1.56
Schaaf *et al.* subject 4	Truncating point mutation	19 yo 5 mo	M	148 cm	−3	47.9 kg	−2.84	21.9	−0.29
Soden *et al.* subject 382	Truncating point mutation	11 yo	F	140 cm	−0.55	60.8 kg	2.02	31	2.35
Soden *et al.* subject 383	Truncating point mutation	8 yo	F	107 cm	−3	16.7 kg	−3	14.6	−0.77
Kanber *et al.* patient 1	Gene deletion	7 yo	F	140 cm	3	41 kg	2.6	20.9	1.92
Buiting *et al.* patient 1	Gene deletion	3 yo	M	102.5 cm	1.86	17.3 kg	1.59	16.5	0.37

BMI, body mass index; cm, centimeter; kg, kilogram; mo, month; yo, years old; yr, years; M, male; F, female.

In contrast to those point mutation cases, individuals with deletions including *MAGEL2*, but not the *SNORD116* cluster, were reported to have much milder phenotypes overall [12,13]. Indeed, while these individuals displayed some motor and developmental delays, with one manifesting mild feeding difficulties, the reporting physicians emphasized that their phenotypic severity was much milder than what is typically seen or reported in PWS and Schaaf-Yang syndrome. Moreover, based on a detailed clinical assessment, no joint contractures, signs of autism, or hyperphagia were reported in either *MAGEL2* whole gene deletion case. The authors of the respective publications had taken this as evidence that loss of *MAGEL2* might not contribute to the pathogenesis of PWS. The idea that a deletion of the entire gene could have a milder effect than a truncating mutation seems paradoxical, and is worth further deliberation. The deletion of the complete paternal copy of the gene and promoter could lead to leaky expression of the maternal copy of the *MAGEL2* gene, as suggested based on studies of mice lacking the paternal *MAGEL2* copy [14]. On the other hand, truncating mutations of the single-exon gene *MAGEL2* would not be expected to cause nonsense-medicated decay of mRNA, but to lead to a truncated protein product, which may have neomorphic effects. 

### 2.1. Schaaf-Yang Syndrome vs. Prader-Willi Syndrome

Since the initial identification of truncating point mutations in *MAGEL2* leading to Schaaf-Yang syndrome, two more studies have identified additional individuals with *MAGEL2* point mutations. Soden *et al.* [15] reported two sisters manifesting decreased fetal movement, neonatal hypotonia, feeding difficulties, developmental delay, and intellectual disability. Both were diagnosed with ASD, and both had contractures of the hands. Hyperphagia, obesity, or PWS facial characteristics were not reported. Mejlachowicz *et al.* [16] identified three fetal siblings manifesting lethal arthrogryposis multiplex congenita (*i.e.*, contractures and decreased fetal movement). Whole exome sequencing revealed the presence of a truncating pathogenic variant in *MAGEL2*, subsequently proven to be present on the paternal allele. One additional, unrelated individual with a *de novo* truncating pathogenic variant in *MAGEL2* was identified by Sanger sequencing of *MAGEL2* in a cohort of individuals with arthrogryposis and/or decreased fetal mobility. This individual manifested microretrognathia, a short neck, and small joint contractures. Moreover, this individual manifested severe hypotonia and died on postnatal day 2 from respiratory distress. Due to the age of this individual, assessment for ASD could not be performed. Pathological examination of other organs and systems were reported as normal.

**Figure 1:**
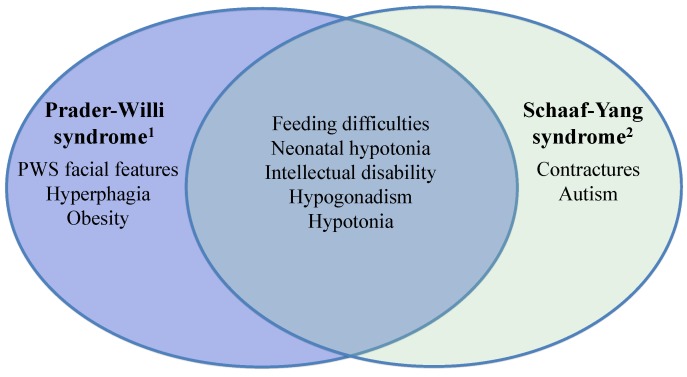
Prader-Willi syndrome and Schaaf-Yang syndrome share common overlapping symptoms, yet have important distinct features. While none of the symptoms listed in this figure may be exclusive to any one condition, the figure attempts to highlight those features that have particularly high prevalence in the respective disorders. Only phenotypes of >50% prevalence for the respective disorder are listed. ^1^, defined by gene deletions in the 15q11-q13 chromosomal region; ^2^, defined by truncating point mutations of the *MAGEL2* gene.

Taken together, the published data suggest a role for *MAGEL2* in PWS and beyond. Both PWS and *MAGEL2*-associated Schaaf-Yang syndrome manifest clinical phenotypes that overlap, and Schaaf-Yang syndrome should be considered an important differential diagnosis of PWS, in particular in the newborn period. Common overlapping phenotypes include neonatal hypotonia, feeding difficulties, hypogonadism, developmental delay and intellectual disability (Figure 1). On the other hand, some clinical features highlight a clinical profile that is unique and distinct from one another. Contractures, which are typically not present in PWS, are seen in a majority of individuals with truncating *MAGEL2* point mutations. Similarly, the prevalence of ASD was recently reported as 27% among individuals with PWS [6]. In contrast, the six individuals with truncating mutations of *MAGEL2*, who were old enough to be assessed, were all clinically diagnosed with ASD based on DSM-IV criteria. As well, hyperphagia and obesity, which are considered hallmark features of PWS, typically seen in nutritional phases 2 and 3 [1], were prevalent in no more than 50% of those individuals with Schaaf-Yang syndrome old enough to be assessed (Table 1).

### 2.2. Animal Models of Magel2 Loss-of-Function

*Magel2* loss of function has been studied in animal models (Table 2). Mice with deletion of most of the *Magel2* gene and its promoter showed a reduction in hypothalamic oxytocin, and a neonatal mortality of 50%, due to suckling defects. Surviving male mice of that strain were shown to manifest deficits in appreciation of social novelty, novel object exploration, and spatial learning and memory [17]. Importantly, both the neonatal lethality, and the social and learning deficits seen in the adult mice could be rescued by early postnatal oxytocin injections [18]. 

**Table 2:** Comparison of reported phenotypic manifestation of *Magel2*-null mouse models.

Wevrick *et al.* [19,20,21,22]	Muscatelli *et al.* [17,18]
Postnatal lethality (10%)	Neonatal lethality (50%)
Decreased pre-wean weight gain	Decreased oxytocin quantity
Increased post-wean adiposity	Decreased suckling activity
Decreased lean mass	Decreased sociability (male mice)
Decreased locomotor activity	Decreased learning and memory (male mice)
Altered appreciation of novelty	
Circadian dysfunction	
Progressive infertility	
Altered olfaction (>24 weeks of age)	

Major phenotypes reported by each group of researchers.

A second *Magel2* loss-of-function mouse model was created by replacing the *Magel2* coding sequence with a *LacZ* reporter, and leaving the original promoter intact. These mice displayed alterations in fat and muscle deposition, neurotransmitter signaling, brain volume, reproduction, and behavior in novel environments [19,20,21,22]. The two described mouse models show a spectrum of manifested phenotypes, but should not be considered definitive or exclusive to either mouse model. Indeed, it may be that a complete phenotypic profile is yet to be reported. Marked phenotypic differences exist between the two mouse models, most dramatically the difference in survival, with a high percentage of neonatal lethality in one model, which is not seen in the other. While increased body adiposity was reported in one, none of the two models recapitulated the hyperphagia and morbid obesity typically seen in human patients with PWS. The low prevalence of the respective phenotypes in human patients with truncating *MAGEL2* mutations may suggest that these phenotypes are, indeed, not attributable to alterations of the *MAGEL2* gene and the respective protein.

### 2.3. USP7 Haploinsufficiency and Its Related Clinical Phenotypes

**Figure 2:**
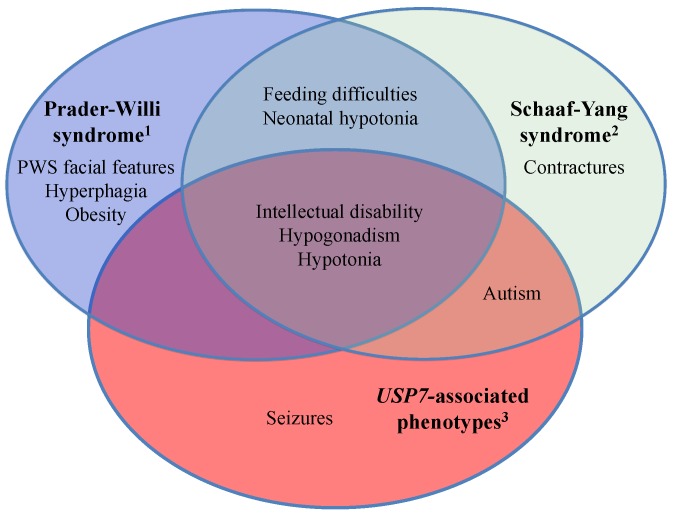
Prader-Willi syndrome, Schaaf-Yang syndrome, and *USP7*-associated disorder represent a spectrum of neurodevelopmental disorders. Shared and distinct clinical features are illustrated. While none of the symptoms listed may be exclusive to any one condition, the figure attempts to highlight those features that have a high (>50%) prevalence in the respective disorders. ^1^, disorder defined by gene deletions in the 15q11-q13 chromosomal region; ^2^, disorder defined by truncating point mutations of the *MAGEL2* gene; ^3^, disorder defined by a deletion or truncating point mutation of the *USP7* gene.

The concept of a molecular and clinical spectrum of disorders is further highlighted by the recent discovery of individuals with loss-of-function mutations in a MAGEL2-interacting protein. Seven individuals were identified to harbor *de novo*, heterozygous deletions/mutations of the gene encoding for ubiquitin-specific protease 7 (USP7). USP7 was shown to interact with MAGEL2 and TRIM27. The three proteins functionally depend on one another, such that alterations in any of these components lead to alterations in endosomal actin assembly, protein recycling, and a subsequent disruption of cellular homeostasis [23]. The seven individuals with *USP7* haploinsufficiency manifested hypotonia, hypogonadism, and developmental delay/intellectual disability, similar to both PWS and Schaaf-Yang syndrome. A high prevalence of ASD, similar to that seen in Schaaf-Yang syndrome, was noted in these individuals. In addition, five out of seven individuals with *USP7* mutation had seizures, indicating a higher prevalence than in PWS (10%–20%) [1] or Schaaf-Yang syndrome (only one described case) [11]. It is important to consider the possibility of an ascertainment bias, such that first reports of new gene-disease associations appear to reflect the more severe end of the actual patient population. However, the phenotypic overlap and differences can be appreciated between the three conditions (Figure 2), and in combination with a detailed molecular understanding of MAGEL2 function, shared pathomechanisms are suggested. At the same time, these recent discoveries suggest that genes encoding for other proteins partaking in WASH ubiquitination and endosomal actin assembly are candidate genes for related neurodevelopmental disorders. 

## 3. Conclusions

As technology progresses and more affected individuals are being identified, the clinical and molecular signatures of the various disorders will become increasingly clear. A greater awareness of these disorders will hopefully lead to increased advocacy, research interest, and ultimately better quality of life for the affected individuals.
